# Rethinking the Place of Qualitative Methods in Behavior Analysis

**DOI:** 10.1007/s40614-022-00362-x

**Published:** 2023-01-12

**Authors:** Victoria Burney, Angela Arnold-Saritepe, Clare M. McCann

**Affiliations:** University of Auckland, Private Bag 92019, Auckland, 1142 New Zealand

**Keywords:** behavior analysis, ABA, qualitative research, methodology, social validity

## Abstract

Single-case design research is pervasive and dominant in the field of behavior analysis (BA). It allows for effective application of behavior change technologies in a wide variety of real-world settings. However, as the field has grown, behavioral scholars have suggested incorporating other methods into the investigator’s toolbox to supplement single-case design. To date, the call to expand beyond using only variations of single-case design as the standard for behavior analytic research has gone largely unheard. Given the need for behavior analytic work to be more closely aligned with consumer and stakeholder needs and priorities, along with a proliferation of practitioners and researchers in the field, now is the time to consider the benefits of qualitative research methods for behavior analysts. In particular, in areas of social validity and in exploring diverse applied topics, qualitative methods may help the field of behavior analysis to achieve greater success with documenting the outcomes from behavior change interventions. The present article explores areas where behavior analysis may benefit from utilizing qualitative methods, namely social validity and breadth of topics for study, and provides examples of the value of qualitative research from other fields. A brief outline of qualitative research is provided alongside consideration of the seven dimensions of applied behavior analysis. In situations where single-case design does not offer behavior analysts sufficient methodological opportunity, qualitative research methods could form a powerful addition to the field of behavior analysis*.*

## The Strengths of Single-Case Design

Since the inception of behavior analysis (BA), single-case design research (e.g., single-case experimental design, subject as own control, or small-n) and BA have been nearly ubiquitous (Kazdin, 2011). Aligned with the goal of translating applications of behavioral principles from the laboratory to real world settings, single-case designs have provided behavior analysts a bridge between experimental and applied research. Consistent with the view that individual differences are key to understanding why behavior changes, and that measuring the individual against their own baseline avoids contamination by intersubject variability (Johnston & Pennypacker, 1993), single-case design offers a tool for demonstrating control over behavior for a single subject (Smith & Little, 2018). An in-depth exploration of single-case design methodology sits outside the scope of this article, but it is widely held that this method of scientific inquiry signals quality and rigor to behavior analysts and is a “gold standard” of behavioral research (Kratochwill et al., 2010).

Combined with research designs which allow for repeated demonstration of effects on the dependent variable, single-case studies can be agglomerated to show the efficacy of behavioral intervention for participants with similar characteristics. If a clinical intervention works for many individuals across repeated applications, there is stronger evidence of its utility for use with individuals with similar characteristics. Recent attention has been paid to calculating effect sizes and meta-analyses of single-case research, to further quantify the effectiveness of behavioral interventions (Dowdy et al., 2021). Indeed, combining single-case demonstrations into a measure of effect-size has reinforced that many treatments based on behavior analytic basic principles and operations represent evidence-based practices for individuals with autism spectrum disorder (Wong et al., 2015), and has promoted increased use of behavior interventions in other domains, including: substance abuse treatment, gerontology, brain injury rehabilitation, pediatric feeding, and occupational safety (Carr & Nosik, 2017).

Single-case design research is highly valued by behavioral researchers, clinicians, and behavioral journals (Shadish & Sullivan, 2011). The method does what it does well, and is here to stay. But, as behavior analysis has developed over the decades, scholars have identified that reliance on single-case design as the only method of scientific investigation may ultimately limit the growth of the field (Friman, 2021). Charting the course of this argument through our own behavioral journals, we see that the limitations of solely using single-case design have been highlighted, and alternatives to this course of action have been signaled. In particular, the utility of qualitative approaches has been considered, but rarely adopted (Schwartz & Olswang, 1996). The current article aims to identify and describe areas where single-case design may not sufficiently serve behavior analysts (e.g., in areas of social validity and in the breadth of topics for investigation) and provide examples of how qualitative methods have helped other fields improve their impact in these domains. Here, the authors urge behavior analysts to augment single-case design research methods with qualitative methods where they can assist in answering behavior analytic questions.

## The Time is Right for Broader Research Methods

More than ever in the history of behavior analysis, the ability to apply flexible, and varied, research methods is paramount. First, because the field is growing, and fast. With the advent and ready adoption of credentialing for behavior analysts, and more recent moves toward licensure in some countries and the majority of states in the United States, the number of practicing (i.e., applied) behavior analysts has grown exponentially, to approximately 55,000 worldwide at the time of writing (compared with almost 20,000 in 2015, and only 6,900 in 2010; Behavior Analyst Certification Board, n.d.). Alongside this proliferation of practitioners there has been a burgeoning of training programs for behavior analysts (Deochand & Fuqua, 2016), an increase in dedicated behavior analytic faculty members at universities, and journals that publish behavior analytic studies. With greater numbers comes increased responsibility to apply our scientific knowledge to the betterment of society. It is important to note that there is a critical need for behavior analytic scholarship to explore topics that contribute to a better world (Kelly et al., 2019), and to relate our “big ideas” to an ever-widening array of social issues (LeBlanc, 2020). An expansion of our data collection and analysis toolkit to include qualitative methods may aid behavior analysts in both basic and applied applications of our science, to solving problems that are of high social significance to clients and stakeholders.

Second, recent criticism from some consumers of behavior analytic services has described behavior analysis as dehumanizing and possibly abusive (DeVita-Raeburn, 2016; Ram, 2020). Centered on the use of ABA within the field of autism, commentary suggests that misinformation may play a role in the shift toward viewing some behavior analytic procedures as harmful to clients and stakeholders (Keenan & Dillenburger, 2018). This demonstrates that efforts of disseminating behavior analysis outside the field have, to date, been largely ineffective. Other explanations of the negative sentiment from some consumers of behavior analytic services suggest behavior analysts have missed the mark on evaluating the social acceptability of interventions, particularly in work with autistic individuals (Leaf et al., 2021). Growing levels of consumer concern highlight an urgent need for the field to respond flexibly in the area of social validity. Increased methodological diversity could provide behavior analysts with effective ways to canvas stakeholder perspectives and better answer questions such as “what is the experience of being involved in behavior analytic intervention?” from the perspective of consumers and the public.

## The Argument for Qualitative Methods

Qualitative methods offer strategies to support a shift toward behavior analytic scholarship that is more responsive to consumer needs and preferences. By providing a methodological paradigm that prioritizes gathering consumer and stakeholder experiences, perspectives, and viewpoints, qualitative methods afford behavior analysts a complementary research tool that can help to solve particular research problems for which single-case designs are not best suited (Ferguson, 1993).

Described as a paradigm that is focused on interpreting meaning and identifying patterns using “words as data” (Braun & Clarke, 2006), qualitative methods allow researchers to examine contextual factors in complex situations, where all the relevant variables cannot easily be teased out (Schwartz & Olswang, 1996). Taking a nonpositivist position (i.e., that meaning is constructed and there is no one “correct” version of reality) qualitative methodologies provide data analysis procedures that are: inductive (e.g., generated by participants rather than researcher hypotheses), constructed (e.g., a product of environment and context), and complicated (e.g., to precisely define, replicate, or control experimentally).

Distinct from single-case design in the level of empirical investigation, degree of control over variables, type of research products, and techniques of analysis; qualitative research permits the asking and answering of questions with a focus on vocal or textual (i.e., verbal) reports and descriptive environmental variables (Čolić et al., 2021). Although qualitative research has vastly different aims and methods to single-case research, it does have congruence with the seven dimensions of applied behavior analysis (Baer et al., 1968), and consideration of the specific ways in which qualitative research aligns with the seven dimensions warrants further exploration.

Qualitative methods prioritize measurement of behavior in the form of verbal report. Although a departure from the quantitative measures routinely used by behavior analysts (primarily: discrete and observable responses), they are no less behavioral in focus. With growing attention paid to nonequivalence relational learning and relational frame theory (e.g., acceptance and commitment therapy; Cihon et al., 2021; Tarbox et al., 2020) verbal report as data is becoming increasingly recognized as critical information to guide the development of ABA interventions that are appropriate and meaningful. Accepting that verbal report provides insight into understanding humans in context and measuring socially important problems (Baer et al., 1987), qualitative methods can both satisfy the behavioral dimension of our applied science and offer techniques for canvasing meaningful areas for application of behavior analytic principles and operations.

Both single-case design and qualitative methods value deep analysis of a single participant as their own control, informing the analytic dimension of ABA. Recognizing the role of context on individual performance, both approaches aim to understand the individual experience, albeit using vastly different data to detect patterns. Both methods apply a cautious approach to generalization: where qualitative research avoids “straying too far from the data” (Sullivan & Forrester, 2018) when describing phenomena and processes that maintain those phenomena, single-case design uses replication or “successive study of additional cases” (Schwartz et al., 1995, p. 96) to draw parallels across participants with similar characteristics, avoiding group or agglomerate data. An understanding that data from an individual subject can sometimes tell us more than aggregated outcome measures closely aligns qualitative and single-case research.

Both methodologies are grounded in applied questions: what does this mean for the person? How does the intervention affect the behavior of the individual? Both aim to discover something about socially important phenomena. Identification of what is valuable, acceptable, and practical is a goal common to single-case and qualitative research with human participants. Taken further, qualitative research is often concerned with understanding problems to be addressed, as conceptualized through the eyes of those who experience the problems (Leko, 2014). Qualitative research offers behavior analysts a way to move beyond a focus on the priorities of the behavior analyst, to those valued by and valid for consumers. Both methodologies are interested in processes rather than products (albeit with a different level of focus on replicability), investigating why behavior occurs in the manner in which it is observed or recorded (Schwartz et al., 1995). In these ways, qualitative research satisfies the applied dimension of behavior analysis.

Qualitative methodologies have advanced in recent years, giving researchers tools that better fit research aims. Past applications of qualitative research have been described as fraught with difficulty and necessary compromise (Smith, 2015). Approaches with strong theoretical or epistemological leanings (such as grounded theory, narrative theory) rarely translated to the aims of psychological or health research. As a result, qualitative methods were applied without comprehensive analysis, or due consideration to theory, leading to what has been described as a “watering down” of research (Thorne, 2016, p. 39). However, shifts in qualitative research have seen a wealth of new methodological approaches developed and refined, supporting examination of questions relevant to behavior analysts without reliance on theory generation or specific epistemological leanings. It is equally true that the growth of qualitative research has afforded increased attention to issues of quality and rigor (Smith, 2015). Tools for assessing the quality of qualitative research have trailed behind other traditions (e.g., quantitative, large-n research; Morse et al., 2002). However, current development of protocols and criteria for evaluating the rigor of qualitative research provides confidence to those who use these methods in their scientific inquiry. For behavior analysts, with a focus on interobserver agreement, treatment fidelity, and other measures of validity (Kazdin, 2011), a shift toward robust assessment of quality provides reassurance that qualitative methods can meet the technological dimension of behavior analysis.

Qualitative research holds as a basic tenet the generation of new findings, and the sharing of findings with interested communities (Braun & Clarke, 2006). This aligns with the behavior analytic goal of generality or generalizability. The ability to share research in ways that will promote, maintain, and appropriately expand the impacts of treatment, has long been at the forefront of behavior analytic research (Baer et al., 1987). By using methods preferred and understood by related disciplines (e.g., nursing, social work, occupational therapy) behavior analysts ensure their findings will “appear in a wide variety of possible environments” (Baer et al., 1968, p. 96) and persist over time. Qualitative methods do not utilize the jargon endemic to ABA, which has been perceived negatively by those outside the field (Critchfield & Doepke, 2018), removing the barrier of translating what behavior analysts know into forms that others can understand. Adopting qualitative methods in appropriate situations, behavior analysts can directly show how or why behavioral interventions “fit” within schools, health settings, and communities (Schwartz et al., 1995), helping to reach broader audiences and improve the application of effective interventions in natural environments.

Given the growth of the field of behavior analysis over the past decades, and developments in qualitative methodologies during this time, it seems important for contemporary behavior analysts to consider how qualitative methods could be applied to empirical questions that are critical to the success of behavior analysis. In particular, increased adoption of qualitative methods where they are the best “fit” for the question, may aid in progressing a socially valid science that can readily investigate a wide variety of topics of interest and relevance to society today (Heward et al., 2022).

## Where Qualitative Methods Could Advance BA: Social Validity

From the beginning, behavior analysts have been concerned with establishing, and measuring, social validity (the degree to which goals, procedures, and effects of behavior analytic input are socially meaningful and relevant to consumers; Kazdin, 1977). Yet, the area of social validity has been fraught with complications in accurate measurement and empirical evaluation (Carr et al., 1999; Ferguson et al., 2018).

In the seminal article “Social Validity: The Case for Subjective Measurement or How Applied Behavior Analysis is Finding Its Heart,” Wolf (1978) posited that social validity created a tension for behavior analysts at the inception of the field because it lent itself to subjective measures rather than tightly defined and controlled experimentation. Wolf identified that variables such as the perceived relevance and usefulness of behavioral intervention by consumers were not amenable to objective definition and measurement, and consequently could not be assessed using single-case design. Rather than discount social validity as subjective and not relevant to ABA, Wolf argued that “if we aspire to social importance, then we must develop systems that allow our consumers to provide us feedback about how our applications relate to their values, to their reinforcers” (p. 213). Even at that early stage, supplementation of traditional behavior analytic measures was proposed, to afford behavior analysts ways “to approach the specific consumer or representatives of the relevant community, and through interviews or ratings, determine much more precisely what the socially significant problems are” (p. 209).

More than a decade later, Schwartz and Baer (1991) identified that, despite widespread agreement on the importance of social validity in behavior analysis, methods for accurately and effectively measuring social validity through research were still lacking. Revisiting the mismatch between traditional behavior analytic procedures for measuring social validity, they suggested that single-case design does not extend to a comprehensive understanding of the field’s social significance or social acceptability, nor does it allow behavior analysts to research how ABA is perceived in wider society. In the absence of strong research methods, they described behavior analysts’ application of “short, simple, bland, undemanding 7-point scales” (p. 192), as biased towards favorable report and thus “socially invalid.” They argued instead that social validity research should use well-designed indirect measures (including self-report using Likert-type surveys) to assess the viability of an intervention to the consumers and stakeholders. They urged behavior analysts to canvas social validity more widely; by asking communities around consumers (as well as consumers themselves) if an intervention is meaningful, appropriate, acceptable and preferred. Explicit in Schwartz and Baer’s argument is the suggestion that, for questions of social validity, single-case designs do not provide all the answers.

Further to this, Schwartz et al. (1995) advocated the use of qualitative methodologies in behavior analytic inquiries, particularly to address questions of social acceptability. Presenting a case study of mixed methods research in inclusive education, they demonstrated that “stronger linkages between qualitative and behavior analytic research methods” provided an avenue to making “behavioral research more responsive to the values and goals of consumers” (p. 97). They posited that qualitative research could help behavior analysts identify the priorities of stakeholders and communities (e.g., parents and school boards), to formalize understanding of relevant variables that can then be empirically tested. Through qualitative methods, acceptability variables would be elucidated before designing an intervention, putting social importance at the forefront of behavior analysis. This inductive approach, Schwartz et al. (1995) argued, could allow recipients of services to set the agenda, rather than relying on what the analyst sees as most important to prioritize for treatment. In this way, qualitative methods provide behavior analysts with tools for avoiding blindness to ideas that are visible to consumers, but not obvious to behavior analysts.

As well as better assessing the social validity of interventions, Schwartz and Olswang (1996) suggested qualitative methods could result in data that are more meaningful to consumers, and answer questions that interest consumers in ways consumers can understand. They reflected that the audience consuming the research outputs must be paramount for behavior analysts as they decide which research methods will lead to compelling findings. It follows that qualitative methods (using words as data) may be more convincing for stakeholders of behavior analytic interventions, and a better way to explore acceptability.

Building from Wolf (1978) and Schwartz and Baer (1991) behavior analysts in the 1990s articulated the call for expanded methodologies, proposing indirect quantitative (questionnaires, surveys; Fawcett, 1991; Kennedy, 1992) and qualitative approaches, to study what is important to consumers (goals; Schwartz, 1991), and how consumers perceive interventions (process and effects; Finney, 1991). Use of research methods “other” than single-case designs were described as potential options to allow the field to learn what is meaningful and valuable for society, rather than exclusively surveying direct recipients (Ferguson, 1993, Schwartz, 1991).

The intervening years saw the application of qualitative methods to social validity in a smattering of single-case design studies, primarily at the intersection of behavioral and special education literatures. Leko (2014) evaluated the acceptability of a phonics-based reading program using teacher interviews coupled with direct observations. Leko noted that qualitative methods in the study offered a different perspective to the complicated picture of treatment acceptability for teachers, arguing qualitative approaches can be “differently informative yet equally valuable” (p. 285) in the pursuit of understanding social validity for clients and stakeholders. In an attempt to measure teacher perspectives of a self-monitoring intervention for students diagnosed with ADHD, Vogelgesang et al. (2016) used questionnaire, semi-structured interview, and journaling methods, with a teacher before and after implementation. Concluding that qualitative data offered insight into “complications” with the intervention that questionnaires alone missed; the authors emphasized the value of mixed-methods analysis of social validity. Nicolson et al. (2020) echoed the call for increased flexibility in assessing social validity. They contend that “having more tools that are designed to examine different aspects of social validity is a necessary step in the evolution of paying more attention to this somewhat neglected dimension of ABA” (p. 760). This reiteration of the need to use other methodologies to examine social acceptability, however, sits alongside the relative paucity of published literature using qualitative or other quantitative methods in behavior analytic journals. There have been a few promising applications of qualitative methods in measuring social validity, but the expansion of methodologies for this purpose has lacked wide adoption by behavior analysts (Snodgrass et al., 2021), and the repeated calls for BA to broaden research methods in studying social validity (Carr et al., 1999; Ferguson et al., 2018) have largely gone unanswered.

## Where Qualitative Methods Could Advance BA: Diverse Topics

Although ABA is often inaccurately described as synonymous with the study of empirically validated approaches for populations with autism, developmental disabilities, and additional learning needs (Axelrod et al., 2012; Normand & Kohn, 2013), the field has long been interested in other areas which pertain to the behavior of humans in context—including addiction (Silverman et al., 2019), obesity (Wilfley et al., 2018), gun safety (Chan & Kirby, 2021), and seatbelt use (Berry & Geller, 1991). Behavior analysts have become increasingly focused on how the field can generate and share findings about human behavior with those who could directly benefit, across settings and situations (Critchfield & Reed, 2017; Freedman, 2015; Heward et al., 2022). When considering the breadth of socially important topics, and the need for BA to “have a say in resolving the most socially relevant problems of our time, such as war, murder . . . disease, public education, and so on” (Vollmer, 2011, p. 34), arguments are emerging that single-case designs could be just one of many methodological tools available to behavioral researchers interested in an ever-widening array of social contexts, and that qualitative investigations may allow behavior analysts to broaden the type of research questions they ask of ever-expanding domains of interest.

In exploring the impact of Baer et al.’s (1968) “seven dimensions” on the current state of behavioral research, Critchfield and Reed (2017) contend that topics that do not lend themselves to precise definition and measurement (i.e., inappropriate under a single-case framework) have been relatively understudied by behavior analysts. Described as “fuzzy concepts”: issues that are socially important and valuable for advancing the field, but poorly defined or with variables not yet fully understood, these domains have largely been underexplored by behavior analysts. This is in part because research in these murky areas faces barriers to publication for not equally demonstrating all seven dimensions of behavior analysis (thought to represent an “overly strict criterion”; Critchfield & Reed, 2017). The reliance on single-case design as the ideal research standard, and the only method which satisfies the “analytic” criteria:

undersells the potential of behavior analysis research to shed light on a wide variety of social problems, discourages interest in problems that do not readily fit into the framework, and supports a too narrow conception of what belongs in applied behavior analysis journals. (Critchfield & Reed, 2017, p. 151)

In their analysis, Critchfield and Reed remind BA researchers of their responsibility to attend to what Baer et al. (1968) call “behaviors which are socially important, rather than convenient for study” (p. 92). Far from ignoring socially important topics, analysts should instead apply other research methods to canvas these topics and move toward definition, measurement, and manipulation of key variables in later analysis. In areas where behavior analysis has yet to fully outline the relevant variables (or where variables are multiple and contradictory) other forms of scientific inquiry, such as description of context through qualitative methods, might offer a way forward. Not only do qualitative methods provide novel ways to conceptualize, design, and conduct research in important areas, they may also support behavior analysts in what Kelly et al. (2019) describe as the “responsibility of every behavior analyst to arrange for contingencies to assure its survival” (p. 449). That is, we need to use a diverse platform of research methods to scope interesting areas of study, and to share newly generated findings in forms consumers are comfortable with, understand, and value (Critchfield & Farmer-Dougan, 2014).

Nicolson et al. (2020) describe the over selectivity of single-case design in ABA literature, which has shaped the field such that other possible methods of inquiry are often not considered by researchers. In addressing the question of what should be done when single-case designs cannot answer all the applied questions behavior analysts, and the wider world, are interested in, Nicolson et al. (2020) suggest that when questions of interest center around client experiences, consumer perspectives and layperson understanding, qualitative research methods are a more cogent fit. Qualitative methods allow exploration of topics that are important to consumers, but ill-defined from an experimental ABA lens, such as “progress,” “inclusion,” and “relationship.”

The contention that space exists for broadening research methods to investigate “fuzzy concepts” has coincided with a groundswell of public interest in cultural and political movements (e.g., Black Lives Matter, Me Too) and compelling societal issues (e.g., terrorism, pandemic management, and public health initiatives; see Conine et al., 2022; Gravina et al., 2020; and Matsuda et al., 2020, for commentary). These are arguably critical areas for behavior analytic investigation but are oftentimes a poor match for research using single-case design, at least in the initial stages of inquiry. Applied behavior analysis, with a unique perspective on socially important human behavior, has a lot to offer in these areas (Dixon et al., 2018; Heward et al., 2022), particularly if there is a willingness to engage with other research perspectives. It is clear that societally relevant research questions that are important to communities within which behavior analysts live and work, do not always lend themselves to single-case design methodology, at least not until more of the variables involved are clear (Critchfield & Reed, 2017; Saini & Vance, 2020). Qualitative research could yield the subjective and rich information necessary to identify variables required for later empirical research in these domains, using single-case or group design.

## How Qualitative Methods Can Help: Expanding Climate Change Research

To exemplify how qualitative methodology can add to diverse avenues of study, the case of qualitative climate change research provides valuable insights. A growing field (owing to the increasingly evident climate change emergency; World Meteorological Organization, 2022) and one historically dominated by quantitative studies, climate change researchers are beginning to incorporate qualitative techniques to answer questions about people’s motivations and reflections on their own behavior, as these relate to actions impacting the environment. In a study on arctic sea-level change in a Norwegian town, Bercht (2021) used an interpretive paradigm and interview data to understand the perspectives and behavior of locals involved in the fisheries industry as they related to sea level change. Bercht identified an apparent contradiction, when participants talked about concerns of climate change but did not adjust their behavior at either home or work; reflecting how each participant positioned themselves within the climate change crisis facing the town. This research illustrated how the varying ways people see themselves within a problem has implications for how individual, or group-level, action plans could be successfully developed and communicated. Bercht argued that “qualitative approaches urgently matter in climate and ocean change research because climate and ocean solutions are conditional on individual and group behaviors . . . institutional settings and governance structures that are impossible to fully understand in solely quantitative studies” (p. 11). In other applications of qualitative methods to the climate change field, McNamara et al. (2020) used interview and focus group data to explore the impact and utility of various community-based climate change initiatives in the Pacific Islands, identifying tangible approaches and actions that promote engagement of locals in designing research, collecting data, and developing climate change interventions. In earlier research, Rao et al. (2019) used a qualitative comparative analysis methodology to explore the role of women’s agency as a variable affecting the effectiveness of climate response programs in Asia and Africa. Without challenging the dominant position of quantitative research in climate change science, current scholars have applied qualitative approaches to better understand the context around the big “problems at hand” (Bercht, 2021) and generate data about experiences and perspectives which could better inform the development of effective climate change strategy.

In climate change research, qualitative methods are gaining prominence alongside the traditional and respected quantitative methods. Qualitative approaches give scholars novel tools for exploring the wider context around an issue, event, or situation, such that practical change can be implemented and evaluated. If applied to the field of behavior analysis, qualitative methods could offer similar benefits; allowing the field to study behavior within increasingly novel contexts, and further elucidate the relevant variables in these contexts, to which the science of behavior could be effective in creating desired change. Taken further, qualitative approaches could be woven into mixed methods investigations, again expanding the scope and applicability of behavior analytic research. Broadening research methods could allow behavior analysts to make greater inroads into interesting, socially relevant, and important domains.

## For the (Un)Convinced: Where to Next?

The current article highlights the value of applying qualitative methods and may compel behavior analysts to adopt qualitative tools, where these afford a convincing methodology for addressing empirical questions. However, the barriers to routine adoption of qualitative research are many and will require careful problem-solving by the behavior analytic community.

Namely, there exists a bias toward funding provision for quantitative studies (in particular randomized control trials and, more recently, single-case designs), where grant funding agencies are more readily convinced by studies that propose well-planned experimental trials or robust single-case designs than qualitatively derived proposals (Morse, 2003). This barrier is not unique to behavior analysis, but it does pose a challenge when calling behavior analysts to use qualitative approaches. Bourgeault (2012) suggests that both the nature of “traditional” qualitative studies (e.g., small in scale, requiring few resources) and myths around the necessary and sufficient conditions for a high-quality qualitative study have resulted in relatively fewer grant applications using qualitative methods. In turn, funding bodies are less familiar with how to evaluate qualitative studies and less confident about potential outcomes from awarding grants to studies using qualitative methods. Although the contextual factors involved in funding provisions are numerous and complicated (and analysis of these variables is warranted), one suggestion for overcoming this hurdle is to coach both applicants and funders in what constitutes a credible qualitative study and appropriate funding application (see Carey & Swanson, 2003, for elaboration).

Related to this, the publication trends evident in behavioral journals (Nicolson et al., 2020) present a contingency whereby behavior analysts who apply qualitative methods in their scholarship may be thwarted by a reduced likelihood of their research being published in reputable, peer reviewed behavioral journals. Although arguably a key variable affecting the adoption of qualitative research methods, this problem is not insurmountable. Recent special editions within ABA journals and editorial calls for diverse scholarship (LeBlanc, 2020) demonstrate that the field is open to, and interested in, a more varied range of perspectives, topics, and research methods in the published literature. It seems that the appropriate motivating operations are present to compel behavior analysts to incorporate qualitative methods into studies published in behavioral journals, when this method is better suited to addressing the research question than single-case design.

Achieving the goal of integrating qualitative methods into behavior science will require that behavior analysts receive rigorous training in qualitative research approaches. Behavior analysts are relatively underinformed about the characteristics of high-quality qualitative methods, what they add to a potential research agenda, and when they should be utilized as the best methodological tool to address the research question or referral concern (Friman, 2021). A recent decision from the Behavior Analyst Certification Board (BACB) to move away from regarding Task Lists as “all-encompassing lists of critical behavior-analytic content” (Behavior Analyst Certification Board, 2022) toward a suggestion of essential skills for behavior analysts (related to, but distinct from, testing content for accreditation), signals that opportunities to widen the methodological training behavior analysts receive in other research approaches are growing. Enthusiastic behavior analysts could work to develop and publish tutorials in qualitative methods, trainings in using qualitative research tools, and broader conceptualizations of research methodology in verified course sequences; all of which would go some way to developing the skills of behavior analysts in applying qualitative approaches.

## Conclusion

As the field of behavior analysis has grown over the past 70 years, attention has been paid to the role of single-case design in defining how, and what, behavior analysts study. Time and again behavior analytic scholars have called for the expansion of research methodologies that behavior analysts can apply to empirical problems. Progress across domains of social validity and diversity of research topics leads the field of behavior analysis to an important conclusion: it is time to expand beyond single-case design research to promote our science. Growth in the field, coupled with rising opposition to ABA in the mainstream, behooves behavior analysts to adopt a range of research methods to best answer a variety of research questions, and to apply the best approach to answer the question, rather than adjusting the topic under study to fit within the more familiar single-case research designs.

In the realm of social validity, where stakeholder perspectives are the key variable of interest, qualitative methods prove a useful addition. Qualitative research provides a methodology to gauge what is important and useful to consumers, and society at large. This could lend credibility to behavior analytic work, and allow behavior analysts to identify when the goals, processes, or products of an intervention could miss the mark for those engaging with the intervention.

Qualitative approaches offer behavior analysts a broader scope to explore interesting and socially relevant topics to which single-case designs do not fit; perhaps because topics require clarification before empirical analyses can be effectively applied, or because key features, phenomenon, and variables are better understood using an inductive, rather than deductive, frame. Inclusion of qualitative research in fields such as climate science demonstrate how qualitative methods can provide behavior analysis another way to increase the reach and scope of our scholarship, to the ultimate benefit of society.

Qualitative methods have evolved rapidly in recent years. They are no longer tied to inflexible philosophical positions or confounded by questionable rigor. Qualitative studies can answer questions that are meaningful to society and hence behavior analysts, with robust and reliable technologies.

This reading of behavioral literature leads us to suggest that the field of behavior analysis could broaden its definition of high quality research to include different methods that answer different, but no less important, questions. Now is the time to expand our horizons and consider a widened scope of research methodologies. In as much as behavior analysts are willing to let the context around behavior dictate selected assessment and intervention approaches, the field should let the context around research questions from the clients’ and the stakeholders’ perspectives drive the selection of research methods, qualitative or quantitative.

This call to consider qualitative methods is not without its challenges. Namely, the apparent lack of funding streams or coherent publication pathways, as well as relative unfamiliarity with qualitative research methods within the behavior analytic field. If now is the time for increased adoption of qualitative methods—and the current authors argue it is—practical tutorials that allow behavior analysts to acquire and become fluent in best practices for using qualitative methods may be an important next step.

## References

[CR1] Axelrod S, McElrath KK, Wine B (2012). Applied behavior analysis: Autism and beyond. Behavioral Interventions.

[CR2] Baer DM, Wolf MM, Risley TR (1968). Some current dimensions of applied behavior analysis. Journal of Applied Behavior Analysis.

[CR3] Baer DM, Wolf MM, Risley TR (1987). Some still-current dimensions of applied behavior analysis. Journal of Applied Behavior Analysis.

[CR4] Behavior Analyst Certification Board. (n.d.). *BACB certificant data*. Accessed: 1 March 2022 at https://www.bacb.com/bacb-certificant-data/

[CR5] Behavior Analyst Certification Board. (2022). *BACB newsletter. *Accessed: 26 May 2022 https://www.bacb.com/wpcontent/uploads/2022/02/BACB_February2022_Newsletter-220207.pdf

[CR6] Bercht AL (2021). How qualitative approaches matter in climate and ocean change research: Uncovering contradictions about climate concern. Global Environmental Change.

[CR7] Berry TD, Geller ES (1991). A single-subject approach to evaluating vehicle safety belt reminders: Back to basics. Journal of Applied Behavior Analysis.

[CR8] Bourgeault IL (2012). Critical issues in the funding of qualitative research. Journal of Ethnographic & Qualitative Research.

[CR9] Braun V, Clarke V (2006). Using thematic analysis in psychology. Qualitative Research in Psychology.

[CR10] Carey MA, Swanson J (2003). Funding for qualitative research. Qualitative Health Research.

[CR11] Carr JE, Austin JL, Britton LN, Kellum KK, Bailey JS (1999). An assessment of social validity trends in applied behavior analysis. Behavioral Interventions.

[CR12] Carr JE, Nosik MR (2017). Professional credentialing of practicing behavior analysts. Policy Insights from the Behavioral & Brain Sciences.

[CR13] Chan, P. E., & Kirby, M. S. (2021). Firearms and home-based ABA services: Considerations for safe practice. *Behavior Analysis in Practice, 15*(2), 553-561. 10.1007/s40617-021-00609-0 10.1007/s40617-021-00609-0 PMC9120302 35692520

[CR14] Cihon, J. H., Ferguson, J. L., Leaf, J. B., Milne, C. M., Leaf, R., & McEachin, J. (2021). Acceptance and commitment training: A review of the research. *European Journal of Behavior Analysis, 1–21*. 10.1080/15021149.2021.1880688

[CR15] Čolić, M., Araiba, S., Lovelace, T. S., & Dababnah, S. (2021). Black caregivers’ perspectives on racism in ASD services: Toward culturally responsive ABA practice. *Behavior Analysis in Practice, 1–10*. 10.1007/s40617-021-00577-5 10.1007/s40617-021-00577-5 PMC8171225 34093981

[CR16] Conine DE, Campau SC, Petronelli AK (2022). LGBTQ+ conversion therapy and applied behavior analysis: A call to action. Journal of Applied Behavior Analysis.

[CR17] Critchfield TS, Doepke KJ (2018). Emotional overtones of behavior analysis terms in English and five other languages. Behavior Analysis in Practice.

[CR18] Critchfield TS, Farmer-Dougan VF (2014). Isolation from the mainstream: Recipe for an impoverished science. European Journal of Behavior Analysis.

[CR19] Critchfield TS, Reed DD (2017). The fuzzy concept of applied behavior analysis research. The Behavior Analyst.

[CR20] Deochand N, Fuqua RW (2016). BACB certification trends: State of the states (1999 to 2014). Behavior Analysis in Practice.

[CR21] DeVita-Raeburn, E. (2016). Is the most common therapy for autism cruel? *The Atlantic* https://www.theatlantic.com/health/archive/2016/08/aba-autism-controversy/495272/

[CR22] Dixon MR, Belisle J, Rehfeldt RA, Root WB (2018). Why we are still not acting to save the world: The upward challenge of a post-Skinnerian behavior science. Perspectives on Behavior Science.

[CR23] Dowdy A, Peltier C, Tincani M, Schneider WJ, Hantula DA, Travers JC (2021). Meta-analyses and effect sizes in applied behavior analysis: A review and discussion. Journal of Applied Behavior Analysis.

[CR24] Fawcett SB (1991). Social validity: A note on methodology. Journal of Applied Behavior Analysis.

[CR25] Ferguson DL (1993). Something a little out of the ordinary: Reflections on becoming an interpretivist researcher in special education. Remedial & Special Education.

[CR26] Ferguson JL, Cihon JH, Leaf JB, Meter SM, McEachin J, Leaf R (2018). Assessment of social validity trends in the *Journal of Applied Behavior Analysis*. European Journal of Behavior Analysis.

[CR27] Finney JW (1991). On further development of the concept of social validity. Journal of Applied Behavior Analysis.

[CR28] Freedman DH (2015). Improving public perception of behavior analysis. The Behavior Analyst.

[CR29] Friman PC (2021). There is no such thing as a bad boy: The circumstances view of problem behavior. Journal of Applied Behavior Analysis.

[CR30] Gravina N, Nastasi JA, Sleiman AA, Matey N, Simmons DE (2020). Behavioral strategies for reducing disease transmission in the workplace. Journal of Applied Behavior Analysis.

[CR31] Heward, W. L., Critchfield, T. S., Reed, D. D., Detrich, R., & Kimball, J. W. (2022). ABA from A to Z: Behavior science applied to 350 domains of socially significant behavior. *Perspectives on Behavior Science*. 33. 10.1007/s40614-022-00336-z PMC9163266 35719874

[CR32] Johnston JM, Pennypacker HS (1993). *Readings for strategies and tactics of behavioral research*.

[CR33] Kazdin AE (1977). Assessing the clinical or applied importance of behavior change through social validation. Behavior Modification.

[CR34] Kazdin AE (2011). *Single-case research designs: Methods for clinical and applied settings*.

[CR35] Keenan M, Dillenburger K (2018). How “fake news” affects autism policy. Societies.

[CR36] Kelly MP, Martin N, Dillenburger K, Kelly AN, Miller MM (2019). Spreading the news: History, successes, challenges and the ethics of effective dissemination. Behavior Analysis in Practice.

[CR37] Kennedy CH (1992). Trends in the measurement of social validity. The Behavior Analyst.

[CR38] Kratochwill, T. R., Hitchcock, J., Horner, R. H., Levin, J. R., Odom, S. L., Rindskopf, D. M. & Shadish, W. R. (2010). *Single-case designs technical documentation*. Accessed 4 April 2022 at http://ies.ed.gov/ncee/wwc/pdf/wwc_scd.pdf

[CR39] Leaf, J. B., Cihon, J. H., Leaf, R., McEachin, J., Liu, N., Russell, N., Unumb, L., Shapiro, S., & Khosrowshahi, D. (2021). Concerns about ABA-based intervention: An evaluation and recommendations. *Journal of Autism and Developmental Disorders, 1–16*. 10.1007/s10803-021-05137-y 10.1007/s10803-021-05137-y PMC9114057 34132968

[CR40] LeBlanc LA (2020). Editor’s note: The power of big ideas. Journal of Applied Behavior Analysis.

[CR41] Leko MM (2014). The value of qualitative methods in social validity research. Remedial & Special Education.

[CR42] Matsuda K, Garcia Y, Catagnus R, Brandt JA (2020). Can behavior analysis help us understand and reduce racism? A review of the current literature. Behavior Analysis in Practice.

[CR43] McNamara KE, Clissold R, Westoby R, Piggott-McKellar AE, Kumar R, Clarke T, Namoumou F, Areki F, Joseph E, Warrick O, Nunn PD (2020). An assessment of community-based adaptation initiatives in the Pacific Islands. Nature Climate Change.

[CR44] Morse JM (2003). A review committee’s guide for evaluating qualitative proposals. Qualitative Health Research.

[CR45] Morse JM, Barrett M, Mayan M, Olson K, Jude S (2002). Verification strategies for establishing reliability and validity in qualitative research. International Journal of Qualitative Methods.

[CR46] Nicolson AC, Lazo-Pearson JF, Shandy J (2020). ABA finding its heart during a pandemic: An exploration in social validity. Behavior Analysis in Practice.

[CR47] Normand MP, Kohn CS (2013). Don’t wag the dog: Extending the reach of applied behavior analysis. The Behavior Analyst.

[CR48] Ram, J. (2020). I am a disillusioned BCBA: Autistics are right about ABA. *NeuroClastic*. Accessed: 16 March 2022 at https://neuroclastic.com/i-am-a-disillusioned-bcba-autistics-are-right-about-aba/

[CR49] Rao N, Mishra A, Prakash A, Singh C, Qaisrani A, Poonacha P, Vincent K, Bedelian C (2019). A qualitative comparative analysis of women’s agency and adaptive capacity in climate change hotspots in Asia and Africa. Nature Climate Change.

[CR50] Saini V, Vance H (2020). Systemic racism and cultural selection: A preliminary analysis of metacontingencies. Behavior & Social Issues.

[CR51] Schwartz IS (1991). The study of consumer behavior and social validity: An essential partnership for applied behavior analysis. Journal of Applied Behavior Analysis.

[CR52] Schwartz IS, Baer DM (1991). Social validity assessments: Is current practice state of the art?. Journal of Applied Behavior Analysis.

[CR53] Schwartz, I. S., & Olswang, L. B. (1996). Evaluating child behavior change in natural settings: Exploring alternative strategies for data collection. *Topics in Early Childhood Special Education, 16*(1), 82–101. 10.1177/2F027112149601600108

[CR54] Schwartz IS, Staub D, Gallucci C, Peck CA (1995). Blending qualitative and behavior analytic research methods to evaluate outcomes in inclusive schools. Journal of Behavioral Education.

[CR55] Shadish WR, Sullivan KJ (2011). Characteristics of single-case designs used to assess intervention effects in 2008. Behavior Research Methods.

[CR56] Silverman K, Holtyn AF, Toegel F (2019). The utility of operant conditioning to address poverty and drug addiction. Perspectives on Behavior Science.

[CR57] Smith, J. (2015). *Qualitative psychology: A practical guide to research methods* (3rd ed.). Sage.

[CR58] Smith P, Little D (2018). Small is beautiful: In defense of the small-N design. Psychonomic Bulletin & Review.

[CR59] Snodgrass MR, Chung MY, Kretzer JM, Biggs EE (2021). Rigorous assessment of social validity: A scoping review of a 40-year conversation. Remedial & Special Education.

[CR60] Sullivan, C., & Forrester, M. (2018). *Doing qualitative research in psychology: A practical guide* (2nd ed). Sage.

[CR61] Tarbox, J., Szabo, T., & Aclan, M. (2020). Acceptance and commitment training within the scope of practice of applied behavior analysis. *Behavior Analysis in Practice, 15*(1), 11-32. 10.1007/s40617-020-00466-3 10.1007/s40617-020-00466-3 PMC8854459 35340381

[CR62] Thorne S (2016). *Interpretive description*.

[CR63] Vogelgesang KL, Bruhn AL, Coghill-Behrends WL, Kern AM, Troughton LC, W. (2016). A single-subject study of a technology-based self-monitoring intervention. Journal of Behavioral Education.

[CR64] Vollmer TR (2011). Three variations of translational research: Comments on Critchfield (2011). The Behavior Analyst.

[CR65] Wilfley DE, Hayes JF, Balantekin KN, Van Buren DJ, Epstein LH (2018). Behavioral interventions for obesity in children and adults: Evidence-base, novel approaches, and translation into practice. The American Psychologist.

[CR66] Wolf MM (1978). Social validity: The case for subjective measurement or how applied behavior analysis is finding its heart. Journal of Applied Behavior Analysis.

[CR67] Wong C, Odom SL, Hume KA, Cox AW, Fettig A, Kucharczyk S, Brock ME, Plavnick JB, Fleury VP, Schultz TR (2015). Evidence-based practices for children, youth, and young adults with autism spectrum disorder: A comprehensive review. Journal of Autism & Developmental Disorders.

[CR68] World Meteorological Organization. (2022). *State of the global climate 2021 (no. 1290)*. Accessed: 24 June 2022 at https://library.wmo.int/doc_num.php?explnum_id=11178

